# Hsa-miR-30a-3p overcomes the acquired protective autophagy of bladder cancer in chemotherapy and suppresses tumor growth and muscle invasion

**DOI:** 10.1038/s41419-022-04791-z

**Published:** 2022-04-21

**Authors:** Thomas I-Sheng Hwang, Po-Chun Chen, Te-Fu Tsai, Ji-Fan Lin, Kuang-Yu Chou, Chao-Yen Ho, Hung-En Chen, An-Chen Chang

**Affiliations:** 1 Division of Urology, Department of Surgery, Shin Kong Wu Ho-Su Memorial Hospital, Taipei, Taiwan; 2 Division of Urology, School of Medicine, Fu-Jen Catholic University, New Taipei, Taiwan; 3 Department of Urology, Taipei Medical University, Taipei, Taiwan; 4 Department of Life Science, National Taiwan Normal University, Taipei, Taiwan; 5 Translational Medicine Center, Shin Kong Wu Ho-Su Memorial Hospital, Taipei, Taiwan; 6 Department of Medical Research, China Medical University Hospital, China Medical University, Taichung, Taiwan; 7 Institute of Traditional Medicine, School of Medicine, National Yang Ming Chiao Tung University, Taipei, Taiwan

**Keywords:** Bladder cancer, Macroautophagy

## Abstract

Bladder cancer (BC) is the second most common urologic cancer in western countries. New strategies for managing high-grade muscle-invasive bladder cancer (MIBC) are urgently required because MIBC has a high risk of recurrence and poor survival. A growing body of evidence indicates that microRNA has potent antitumorigenic properties in various cancers, and thus, therapeutic strategies based on microRNA may show promising results in cancer therapy. Analysis of The Cancer Genome Atlas (TCGA) database indicated that hsa-miR-30a-3p is downregulated in human BC. Our in vitro investigation demonstrated that hsa-miR-30a-3p suppresses the expression of matrix metalloproteinase-2 (MMP-2) and MMP-9 and reduces the cell invasive potential of BC cells. Furthermore, hsa-miR-30a-3p directly targets ATG5, ATG12, and Beclin 1; this in turn improves the chemosensitivity of BC cells to cisplatin through the repression of protective autophagy. In a tumor-xenograft mice model, hsa-miR-30a-3p suppressed muscle invasion. Cotreatment with hsa-miR-30a-3p enhanced the antitumor effect of cisplatin in reducing tumor growth in BC. The current study provides a novel strategy of using hsa-miR-30a-3p as an adjuvant or replacement therapy in future BC treatment.

## Introduction

Bladder cancer (BC), a common urological malignancy worldwide, is the eighth most common cause of cancer-related death among men [1]. BC is classified as low-grade non–muscle-invasive bladder cancer (NMIBC) or high-grade muscle-invasive bladder cancer (MIBC) [2], and 50% of patients with MIBC have a higher incidence of distant metastasis and poor prognosis than those with NMIBC do at diagnosis [3, 4]. Although treatment for MIBC has been developed, the associated morbidity and mortality remain significant concerns [5]. To overcome these urgent challenges, identifying novel biomarkers and effective therapeutic options are warranted in BC treatment.

Thus far, the dual roles of autophagy, namely cancer progression and prevention, remain unclear [6]. Our prior study identified that autophagy is aberrantly activated in human BC [7]. A high basal level of autophagic flux in BC cells functions as a “protective” mechanism for cancer cell survival when exposed to chemotherapeutic drugs [8]. Therefore, interrupting protective autophagy is a promising solution that can increase the sensitivity of BC to therapeutic interventions. Emerging research indicates that autophagy also participates in multiple steps of tumor progression, including cancer cell invasion [9], epithelial–mesenchymal transition [10], cancer stem cell differentiation [11], anoikis resistance [12], tumor dormancy [13], and eventually distant metastasis [14]. Thus, a better understanding of the cellular and functional correlation of autophagy in advancing BC will help translate laboratory research into clinical applications.

Since 2018, two small-interfering RNA drugs, namely patisiran and givosiran, have been approved by the U.S. Food and Drug Administration; these drugs have increased the number of clinically valuable applications of drugs using microRNA (miRNA) [15, 16]. Typically, miRNA is a short, mature, functional transcript of approximately 22 nucleotides and can pleiotropically target genes. Numerous miRNAs have been reported to have potent antitumorigenic properties of circumventing multidrug resistance in cancer chemotherapy [17], suppressing cell invasiveness mediated through epithelial–mesenchymal transition [18], and functioning as immune-modulatory miRNAs to regulate cancer immune evasion [19], all of which contribute to the increasing interest of researchers in targeting miRNAs in cancer therapy [20]. In our previous study, we identified a panel of 49 differentially expressed miRNAs in BC tissue and adjacent normal bladder tissue by using a miRNA microarray. Among 19 miRNAs that specifically decreased in BC, hsa-miR-30a may serve as a potential tumor-suppressor miRNA [21]. Yao et al. demonstrated that hsa-miR‐30a expression decreased in BC patients with short overall survival (OS) and disease‐free survival periods [22]. However, the cellular mechanisms by which hsa-miR-30a sabotages BC progression are ill defined. Identifying the antitumorigenic properties of hsa-miR-30a in BC and developing advanced miRNA-based therapy can be powerful tools for the prevention and treatment of BC.

Here, we found that hsa-miR-30a, which is remarkably downregulated in BC tissue, not only effectively suppresses the autophagy related (ATG) 5, ATG12, and Beclin 1 proteins but also inhibits matrix metalloproteinase (MMP)2 and MMP9-mediated cell migration and invasion in BC. Moreover, we found that cell transfection with an hsa-miR-30a-3p mimic blocks protective autophagy through ATG5, ATG12, and Beclin 1 suppression, which in turn increases cancer cell sensitivity to chemotherapeutic interventions in vitro and reduces tumor growth and muscle invasion in vivo. Our evidence indicates that hsa-miR-30a-3p enhances chemotherapy through the inhibition of protective autophagy and can be effective in the treatment of MIBC through the suppression of MMP2 and MMP9-regulated cell invasiveness.

## Materials and methods

### Cell culture

Human BC cell lines (5637 and T24) were obtained from Bioresource Collection and Research Center (Hsinchu, Taiwan) and incubated at 37 °C under 5% CO_2_. The 5637 and T24 cells were cultured in RPMI-1640 and McCoy’s 5A medium, respectively (Gibco, Thermo Fisher Scientific, Inc., MA, USA). All culture media were supplemented with 10% fetal bovine serum (FBS), 2 mM GlutaMAX-1, 100 U/mL penicillin, and 100 µg/mL streptomycin.

### Database

The Cancer Genome Atlas Urothelial Bladder Carcinoma (TCGA-BC) dataset available from the Gene Expression Profiling Interactive Analysis 2 (GEPIA2) web server (http://gepia2.cancer-pku.cn/#index) was used to analyze the correlations among MMP2, MMP9, ATG5, ATG12, and Beclin 1 expression. The OS rate between BC patients with high and low MMP9 and miR-30a expression was detected using OncoLnc (http://www.oncolnc.org/). The Gene Expression database of Normal and Tumor tissues (GENT2; http://gent2.appex.kr/gent2/) tool was used to observe the levels of MMP2 and MMP9 expression in the clinical stage in patients with BC.

### Transfection of miRNA mimic

MISSION synthetic negative control mimic, hsa-miR-30a-3p mimic, and hsa-miR-30a-5p mimic were purchased from Sigma-Aldrich/Merck KGaA (Darmstadt, Germany). Viromer BLUE (Lipocalyx GmbH, Halle, Germany), a miRNA transfection reagent, was used to transfect control mimic (25 nM), miR-30a-5p mimic (25 nM), or miR-30a-3p mimic (25 nM) into BC cells. After 24 h, cell samples were evaluated for indicated protein expression and their migration and invasion abilities.

### Resazurin-based cell viability assay

Transfection of BC cells with the control mimic (25 nM), miR-30a-5p mimic (25 nM), or miR-30a-3p mimic (25 nM) followed by with or without cisplatin (2 μM) treatment for 24 h. After the treatment, resazurin-based cell viability was analyzed according to the manufacturer’s protocol (Biotium, Fremont, CA, USA). The fluorescent signal was measured by using a Varioskan LUX multimode microplate reader (Thermo Fisher Scientific, Waltham, MA, USA).

### Clonogenic survival assay

The BC cells were seeded on six-well plate. After the indicated treatment, the colonies were allowed to grow for 7 days, then fixed with 3.7% formaldehyde, stained with crystal violet (w/v). The colonies were quantified by measuring the absorbance of crystal violet extraction with 10% acetic acid.

### Establishment of pri-hsa-miR-30a stable cells

The pri-hsa-miR-30a sequence was constructed in pLenti-III-micro-GFP vector by Applied Biological Materials, Inc. (British Columbia, Canada). A lentivirus containing pri-hsa-miR-30a expression vector was prepared according to the standard protocol (ALISA Bioscience, Taichung, Taiwan). To establish the pri-hsa-miR-30a stable cell, T24 cells were seeded in a six-well dish and then infected with the lentivirus (multiplicity of infection = 10). After 48 h, 1 μg/mL of puromycin was added to select pri-hsa-miR-30a-expressing stable cells. All surviving cells were selected, and clonal cell populations were expanded.

### Luciferase reporter assay

The DNA fragments containing three-prime untranslated regions (3′-UTRs) of ATG5, ATG12, and Beclin 1 targeted by miR-30a-5p or -3p were constructed into pmiR-GLO dual-luciferase miRNA target expression vector (Promega, WI, USA) by MDBio, Inc. (Taipei, Taiwan). The BC cells were transfected with empty vector (pmiR-GLO) or 3′-UTR reporter constructs (1 μg/μL) by using Viromer RED (Lipocalyx GmbH, Halle, Germany) for 24 h followed by miR-30a-5p or -3p mimic treatment. The activities of firefly and Renilla luciferase were measured using a Dual-Luciferase Kit (Promega, WI, USA). Relative activity was expressed as firefly/Renilla luciferase ratio.

### Transwell migration and invasion assay

Transwell inserts in 24-well dishes (8-µm pore size; Costar, NY, USA) were used to analyze cell migration and invasion. For invasion assays, Transwell inserts were precoated with 30 µL Matrigel basement membrane matrix (BD Biosciences, CA, USA) for 30 min. Human BC cells (2 × 10^4^) in 200 µL serum-free medium were seeded in Transwell inserts, and a 300-µL medium containing 1% FBS was placed in the lower chamber. The migrated cells were stained with 0.05% crystal violet for 30 min and then imaged under ×200 magnification by using an Eclipse Ti2 microscope (Nikon, Tokyo, Japan).

### Western blot analysis

The electrophoresis and transfer methods were described in our previous study [23]. Membranes were incubated with primary anti-MMP-2/9 (GeneTex, CA, USA), ATG5/12 (Abcam, CB, UK), Beclin 1 (Cell Signaling, MA, USA), LC3-I/II (GeneTex, CA, USA), cleaved-caspase-3 (Abcam, CB, UK), cleaved poly (ADP-ribose) polymerase (PARP; Cell Signaling, MA, USA), anti-β-actin (Merck, Darmstadt, Germany), and anti–glyceraldehyde 3-phosphate dehydrogenase (Abcam, CB, UK) antibodies at 4 °C overnight. Furthermore, they were incubated with the appropriate horseradish peroxidase-conjugated anti-rabbit antibody (Cell Signaling, MA, USA) or anti-mouse antibody (Sigma-Aldrich/Merck KGaA, Darmstadt, Germany) at 37 °C for 1 h. Enhanced chemiluminescence was then added to the blots, which were imaged using a ChemiDoc-It Imaging System (UVP Inc., CA, USA).

### Quantitative real-time polymerase chain reaction

Total RNA samples were isolated from BC cells and converted to cDNA using Magic RT cDNA synthesis kit (Bio-genesis Technologies, lnc., Taipei, Taiwan). Quantitative reverse transcriptase PCR (qRT-PCR) analyses were carried out using a Taqman one-step PCR Master Mix (Applied Biosystems, CA, USA) and conducted in triplicate on a StepOnePlus sequence detection system (Thermo Fisher Scientific, MA, USA), as described previously [24].

### Flow cytometry

The BC cells were treated with cisplatin and hsa-miR-30a-3p mimic for 24 h, and the apoptotic cells were detected using the fluorescein isothiocyanate Annexin V apoptosis detection kit (BD Biosciences, NJ, USA), according to the manufacturer’s instructions. The populations with Annexin V^−^/PI^−^, Annexin V^+^/PI^−^, Annexin V^+^/PI^+^, and Annexin V^−^/PI^+^ cells showed live, early apoptotic, late apoptotic, and necrotic cells that were estimated using the Accuri C5 flow cytometer (BD Biosciences, NJ, USA), and the resulting data were analyzed using CellQuest Pro software (BD Biosciences, NJ, USA).

### Caspase-3 activity

The BC cells were cotreated with cisplatin and hsa-miR-30a-3p mimic for 24 h. The levels of caspase-3 activity were then directly measured using an EnzChek Caspase-3 assay kit (Thermo Fisher Scientific; MA, USA) according to the manufacturer’s instructions. The fluorescence intensity of R110 was then measured with an excitation of 485 nm and emission of 535 nm by using the Varioskan LUX multimode microplate reader (Thermo Fisher Scientific).

### Intravesical administration of lenti-miR-30a-3p and cisplatin in vivo

All procedures of the animal study were approved by the Institutional Animal Care and Use Committee and performed according to the Guidelines of Animal Experimentation of Shin Kong Wu Ho-Su Memorial Hospital.

The intravesical BC animal model was constructed using 6-week-old male severe-combined-immunodeficient (SCID) mice (*N* = 6). In brief, 0.125% trypsin with sterile Dulbecco’s modified Eagle’s medium base medium (100 μL) was injected into the bladder using a catheter to traumatize the bladder for 30 s, followed by an injection of 100 μL phosphate-buffered saline to flush the solution from the bladder. The UMUC3-Luc cells (1 × 10^6^) were added to the complete medium/Matrigel (1:1) mix and were introduced to the bladder through the urethra. After bladder tumor stabilization for 1 week, lentiviral particles carrying miR-30a-3p (Lenti-miR-30a-3p; 100 μL), which was obtained from ABM (CA, USA), were first intravesically delivered into mice for 1 h followed by cisplatin treatment (2.5 mg/kg) for another 1 h the next day. Intravesical therapy was provided twice weekly, and tumor size was monitored using a bioluminescence imaging system (PerkinElmer, IVIS Spectrum Imaging System) every week. After 4 weeks, tumor tissues were obtained to determine protein expression.

### Immunohistochemistry

Paraffin-embedded tissues were obtained from the in vivo animal model and then stained with anti-Ki67 (Abcam, CB, UK), anti-LC3-II (GeneTex, CA, USA), anti-MMP2 and anti-MMP9 (GeneTex, CA, USA), anti-Beclin 1 (Cell Signaling, MA, USA), and anti-ATG5/12 (Abcam, CB, UK) according to the method described in our previous study [23]. Immunohistochemistry (IHC) results were scored by taking into account the percentage of tumor tissue with indicated protein staining (0–100%) by Image J software and the intensity of the staining (0, negative; 1, weak; 2, moderate; 3, strong) conducted by three independent pathologists, providing a final score ranging from 0 to 300.

### Statistics

All experiments were performed at least three times, each time in triplicate. Two samples were statistically compared using Student’s *t-*test. One-way analysis of variance followed by Bonferroni’s post hoc comparison test were used to compare the means of more than two groups. Analytical graphs were plotted using GraphPad Prism 9 software. The results are presented as means ± standard deviations. Statistical significance was indicated if *P* < 0.05.

## Results

### Low-level hsa-miR-30a expression is observed in patients with BC

**Fig. 1:**
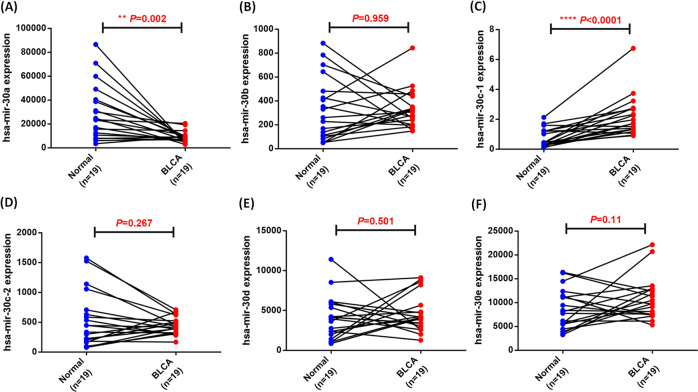
Expression of hsa-miR-30 family members in the TCGA-BC dataset. (**A**–**F**) In the paired samples of BC tissues and adjacent normal tissues (*N* = 19), the expression levels of hsa-miR-30 members were determined through a paired *t*-test. *P < 0.05, **P < 0.01, ***P < 0.001, and ****P < 0.0001 relative to the normal group.

The hsa-miR-30 family consists of hsa-miR-30a, hsa-miR-30b, hsa-miR-30c-1, hsa-miR-30c-2, hsa-miR-30d, and hsa-miR-30e, all of which share the same seed sequence but have different compensatory sequences near the 3′ end, allowing the miR-30 family members to target different genes and pathways to perform diverse biological functions [25]. The analysis of TCGA-BC datasets revealed that hsa-miR-30 members are expressed in patients diagnosed with BC. Hsa-miR-30a levels were lower in the BC tissue than in the normal tissue (Fig. 1A); no other significant between-group differences were observed in terms of miR-30 members (Fig. 1B–F). Patient tissue analysis revealed the unique clinical importance of hsa-miR-30a in BC, which is different from other hsa-miR-30 members. Furthermore, we analyzed the expression pattern of hsa‑miR‑30a in BC based on patient sex, ethnicity, nodal metastasis status, and OS, and the resulting data found that hsa‑miR‑30a expression was more enhanced in Caucasians than in African‑Americans (Fig. S1B); however, no correlations were found among hsa-miR-30a, patient sex, nodal metastasis status, and OS in BC (Fig. S1A, C, D). Such findings indicate that hsa‑miR‑30a expression varies according to genetic differences.

### Hsa-miR-30a-3p suppresses MMP2 and MMP9 expression and cell invasiveness in BC

**Fig. 2:**
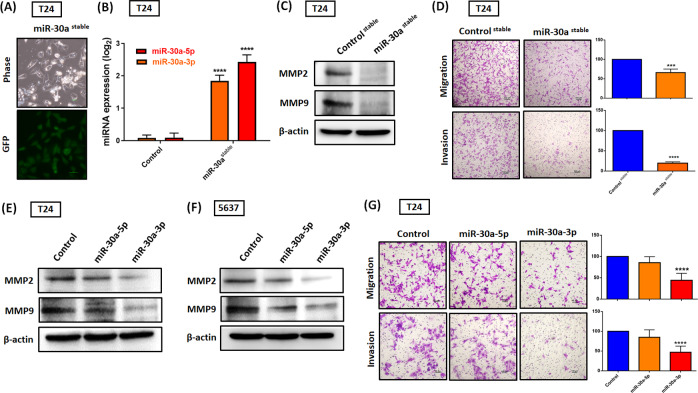
Hsa-miR-30a-3p reduces MMP2, MMP9 expression and cell invasiveness in BC. **A**, **B** Stable cells continuously expressing pri-hsa-miR-30a were confirmed through GFP imaging and hsa-miR-30a-3p or -5p expression (*N* = 6). **C** MMP2 and MMP9 protein expression were evaluated using a western blot assay (*N* = 3). **D** The migration (*N* = 5) and invasion abilities (*N* = 4) of miR-30a stable cells were measured using Transwell assay. **E**–**G** BC cells (T24 and 5637) were transfected with control mimic (25 nM), miR-30a-5p mimic (25 nM), or miR-30a-3p mimic (25 nM) for 24 h; the MMP2 and MMP9 protein expression (*N* = 3) and cell migration (*N* = 10) and invasion abilities (*N* = 7) were analyzed through western blot assay and Transwell assay, respectively. All data are expressed as means ± SDs in triplicate samples. *P < 0.05, **P < 0.01, ***P < 0.001, and ****P < 0.0001 relative to the control group.

The role of MMP2 and MMP9 in tumorigenesis and invasion is documented [26]. Given the importance of these mechanisms, we investigated whether hsa-miR-30a-3p functions as a tumor-suppressor miRNA through MMP2 and MMP9 suppression in BC. Stable cells that constitutively expressed pri-miR-30a-GFP, which was established and confirmed through fluorescence imaging (Fig. 2A) and qRT-PCR assay (Fig. 2B), were observed to downregulate MMP2 and MMP9 expression (Fig. 2C and Fig. S2A) as well as inhibit cell migration and invasiveness (Fig. 2D). Furthermore, the hsa-miR-30a-3p mimic, not the hsa-miR-30a-5p mimic, significantly decreased MMP2 and MMP9 expression (Fig. 2E, F and Fig. S2B), cell migration and invasion (Fig. 2G).

**Fig. 3:**
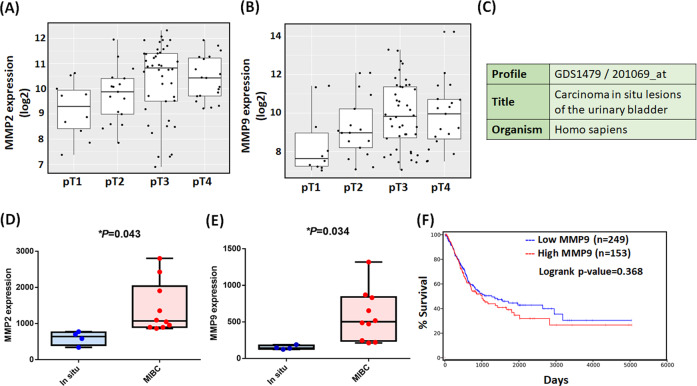
MMP2 and MMP9 mRNA expression in BC tissue. **A**, **B** The GENT2 web server was used to analyze correlations between MMP2 and MMP9 gene expression and primary tumor status. **C**–**E** The BC profiles (GDS1479) in GEO confirmed the MMP2 and MMP9 mRNA level in carcinoma in situ tissues (*N* = 4) and MIBC tissues (*N* = 10). **F** OS rates in patients with BC with high (*N* = 153) and low (*N* = 249) MMP9 expression levels were analyzed using the OncoLnc web server. All data are expressed as means ± SDs in triplicate samples. *P < 0.05, **P < 0.01, ***P < 0.001, and ****P < 0.0001 relative to the pT1, In situ, or Low MMP9 group.

Next, we explored the clinical importance of MMP2 and MMP9 in human BC patients. MMP2 and MMP9 was highly associated with primary tumor status (T) (Fig. 3A, B and Table 1). Microarray analysis of BC profiles (GDS1479) in GEO confirmed that MMP2 and MMP9 mRNA level in MIBC tissues was remarkably increased compared with that in carcinoma in situ tissues (Fig. 3C–E). To assess the prognostic value of MMP2 and MMP9 expression in patients with BC, we investigated the associations between MMP2 and MMP9 expression and OS. In our previous study, the high MMP2 expression observed in patients with BC was correlated with poor OS [27]; however, MMP9 showed no such effect (Fig. 3F). These results suggest that MMP2 and MMP9 overexpression occurs in BC and is positively correlated with muscle invasion, indicating that MMP2 and MMP9 is an excellent target for enhancing BC treatment. We concluded that hsa-miR-30a-3p significantly suppresses MMP2 and MMP9 expression and cell invasiveness in BC, which may become a potential tool of miRNA-based cancer therapy targeting MIBC.

**Table 1:** Significant results of association between MMPs and clinical tumor status based on a two-sample *t-*test.

MMPs	Tissue	*P* value	Log2FC
MMP2	pT4 vs. pT2	0.060	−0.647
	pT4 vs. pT3	0.791	−0.081
	pT4 vs. pT1	0.009**	−1.229
	pT2 vs. pT3	0.112	0.566
	pT2 vs. pT1	0.203	−0.582
	pT3 vs. pT1	0.014*	−1.148
MMP9	pT4 vs. pT2	0.281	−0.607
	pT4 vs. pT3	0.897	0.062
	pT4 vs. pT1	0.043*	−1.452
	pT2 vs. pT3	0.163	0.670
	pT2 vs. pT1	0.217	−0.845
	pT3 vs. pT1	0.024*	−1.515

*P < 0.05, **P < 0.01

### Hsa-miR-30a-3p significantly inhibits in vivo tumor growth and muscle invasion

**Fig. 4:**
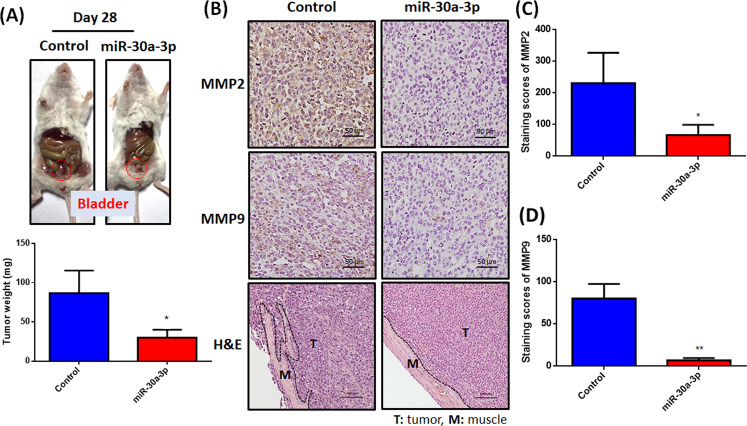
Hsa-miR-30a-3p prevents tumor growth and muscle invasion in tumor-xenograft mice model. (**A**) UMUC3 cells were intravesically injected into SCID mice. Starting 1 week after tumor stabilization, the mice received twice-weekly lenti-miR-30a-3p plasmid. After 28 days, the tumor weights were recorded manually and quantified (N = 3). (**B–D**) IHC staining revealed MMP2 (N = 3) and MMP9 (N = 3) expression in murine tumor tissues. H&E staining was used to perform muscle invasion areas (T: tumor and M: muscle) (N = 3). All data are expressed as means ± SDs in triplicate samples. *P < 0.05, **P < 0.01, ***P < 0.001, and ****P < 0.0001 relative to the control group.

To validate the potential of hsa-miR-30a-3p as a novel miRNA-based therapy in the suppression of tumor growth in vivo, lenti-miR-30a-3p was intravesically administered to tumor-xenograft mice twice a week. Tumors in the lenti-miR-30a-3p-treated group were smaller compared with those in the non–mRNA-treated group (Fig. 4A). Notably, lenti-miR-30a-3p therapy suppressed MMP2 and MMP9 expression and muscle invasion in mice with BC (Fig. 4B–D). Clearly, hsa-miR-30a-3p was a powerful tool that suppresses BC tumor growth and muscle invasion in in vivo analysis.

### Hsa-miR-30a widely targets ATG5, ATG12, and Beclin 1

**Fig. 5:**
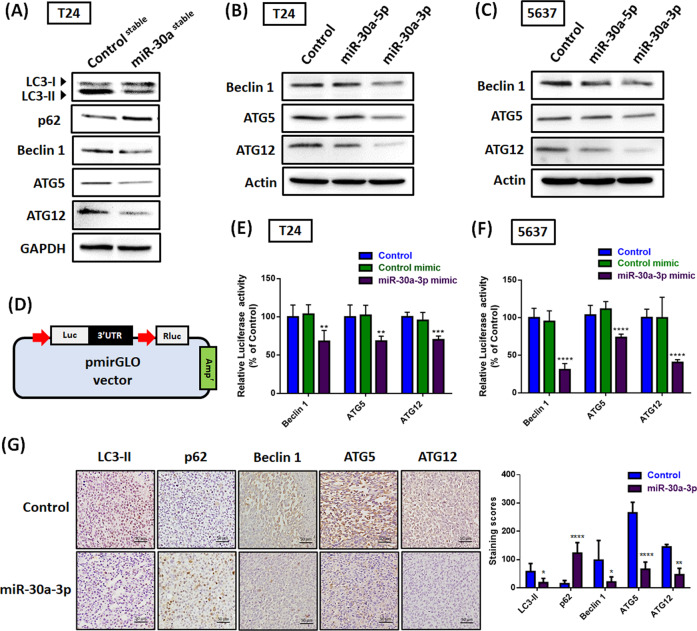
Hsa-miR-30a-3p inhibits ATG5, ATG12, and Beclin 1 expression. **A** LC3-II, p62, ATG5/12, and Beclin 1 protein expression in stable cells were measured through western blot assay (*N* = 3). **B**, **C** Transfection of BC cells (T24 and 5637) with the control mimic (25 nM), miR-30a-5p mimic (25 nM), or miR-30a-3p mimic (25 nM) for 24 h; the levels of ATG5/12 and Beclin 1 protein expression were analyzed through western blot assay (*N* = 3). **D** Schematic 3′-UTR representation of human ATG5/12 and Beclin 1 containing the hsa-miR-30a-3p-binding site in pmiR-GLO luciferase vector. **E**, **F** The BC cells were cotransfected with indicated-3′-UTR plasmid (1 μg/μL) and hsa-miR-30a-3p mimic (25 nM) for 24 h; relative luciferase/Renilla activities were then measured (*N* = 3). **G** Histologic sections of murine tumor were immunostained with LC3-II, p62, ATG5/12, and Beclin 1 (*N* = 3). All data are expressed as means ± SDs in triplicate samples. *P < 0.05, **P < 0.01, ***P < 0.001, and ****P < 0.0001 relative to the control group.

High basal levels of autophagic flux in BC protect cancer cells against chemotherapeutic intervention [8]. To evaluate whether hsa-miR-30a serves as a novel autophagy inhibitor in BC cells, we evaluated several miRNA target prediction programs (i.e., TargetScan, miRDB, and miRNA.org) and confirmed that hsa-miR-30a-3p and -5p may directly target the 3′-UTR regions of ATG5/12 and Beclin 1. We found miR-30a-stable cells reduced the conversion of LC3-I to LC3-II and induced p62 accumulation, indicating a deficiency of autophagy (Fig. 5A). Decreases in total ATG5/12 and Beclin 1 protein were also observed in miR-30a-stable cells (Fig. 5A). Moreover, transfection of the wild-type BC cell lines T24 and 5637 with the hsa-miR-30a-3p mimic revealed that the hsa-miR-30a-3p mimic suppresses ATG5/12 and Beclin 1 more than the hsa-miR-30a-5p mimic does (Fig. 5B, C). These results are consistent with the aforementioned observation that hsa-miR-30a-3p, but not hsa-miR-30a-5p, has a superior inhibition ability in BC (Fig. 2).

To prove that hsa-miR-30a-3p directly binds to the 3′-UTR of ATG5/12 and Beclin 1, which may lead to the inhibition of target mRNA translation, we constructed the hsa-miR-30a-3p-binding sites of ATG5/12 and Beclin 1 3′-UTR luciferase plasmids (Fig. 5D). Cell transfection with the hsa-miR-30a-3p mimic decreased luciferase activity in the ATG5/12 and Beclin 1 3′-UTR plasmid, but the control mimic did not affect the luciferase activity (Fig. 5E, F). Histopathological examination revealed lower levels of LC3-II, ATG5/12, and Beclin 1 expression and higher levels of p62 expression in the tumor tissue of mice treated with hsa-miR-30a-3p compared with those that were not (Fig. 5G). In sum, hsa-miR-30a-3p binds to the 3′-UTR of ATG5/12 and Beclin 1 to suppress mRNA translation, resulting in the downregulation of the autophagic flux of BC.

### ATG5/12 and Beclin 1 are not required for MMP2 and MMP9-mediated cell invasiveness in BC

**Fig. 6:**
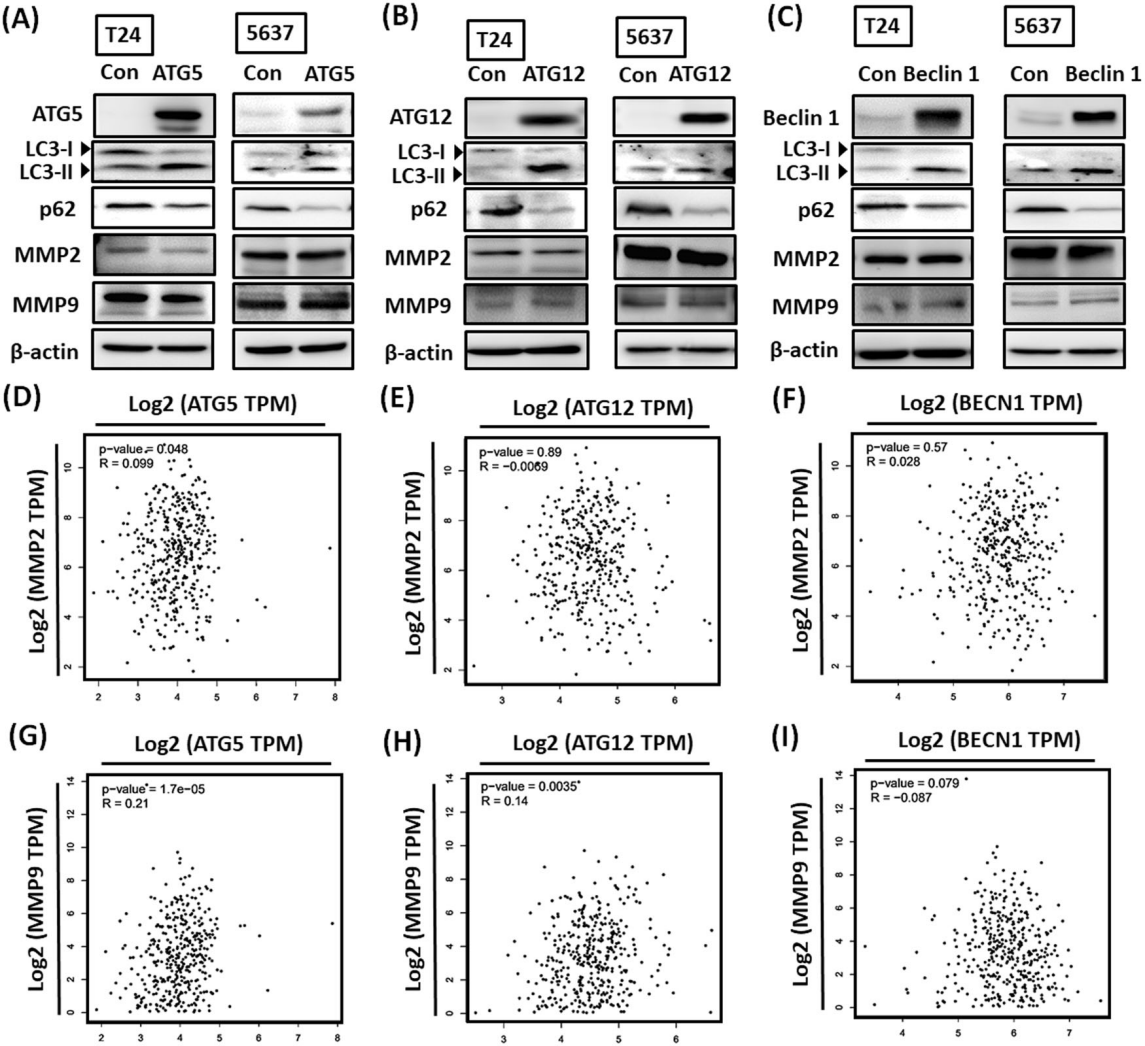
ATG5/12 and Beclin 1 are not upstream mediators of MMP2 and MMP9 in BC cells. **A**–**C** BC cells were transfected with ATG5, ATG12, or Beclin 1 expression plasmid (1.5 μg/μL) for 24 h. The levels of indicated protein expression were examined through western blot assay (*N* = 3). **D**–**I** Correlations among ATG5/12, Beclin 1, MMP2, and MMP9 expression levels in human BC samples analyzed using the GEPIA2 web server. All data are expressed as means ± SDs in triplicate samples. *P < 0.05, **P < 0.01, ***P < 0.001, and ****P < 0.0001 relative to the control group.

Autophagy confers pro-oncogenic effects, namely in promoting tumor cell invasiveness, modulating tumor cell dormancy, helping tumor cells to evade immune surveillance, and eventually contributing to metastasis [14]. For a deeper understanding of whether autophagy is involved in MMP2 and MMP9-mediated cell invasiveness in BC, we established T24 and 5637 cells overexpressing ATG5/12 and Beclin 1. ATG5/12 and Beclin 1 overexpression in cancer cells promoted LC3-II expression and inhibited p62 accumulation, indicating upregulation of autophagic flux (Fig. 6A–C). However, MMP2 or MMP9 had no effect upon ATG5/12 and Beclin 1 overexpression (Fig. 6A–C). To further validate the correlations among ATG5/12, Beclin 1, MMP2, and MMP9 in BC, we analyzed urothelial bladder carcinoma datasets on the GEPIA2 web server. No clinical relations were revealed among these five genes in BC tumors (Fig. 6D–I). In sum, we confirmed that ATG5/12- and Beclin 1-related autophagy is not an upstream mediator of MMP2 and MMP9 in BC cells.

### Hsa-miR-30a-3p attenuates cisplatin-induced protective autophagy and advances chemosensitivity in BC cells

**Fig. 7:**
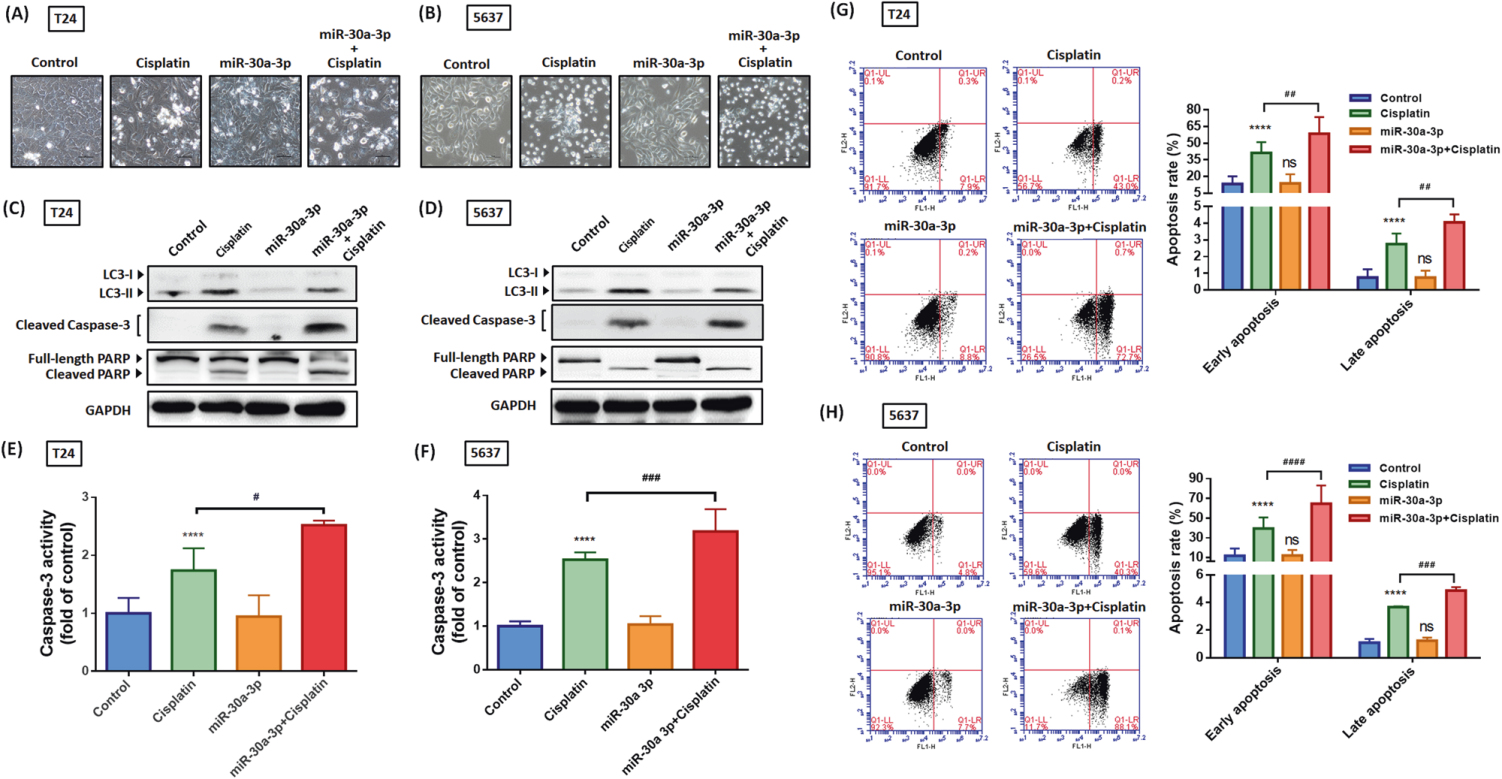
Hsa-miR-30a-3p enhances chemosensitivity in BC cells. **A**, **B** Mono-administration of BC cells with cisplatin (20 μM) or miR-30a-3p mimic (25 nM) or in combination for 24 h; cell morphology under a bright field microscope was captured (*N* = 3). **C**, **D** The BC cells were treated as described in (**A**). The protein levels of LC3-II, cleaved-caspase-3, and cleaved PARP were measured through western blot assay (*N* = 4). **E**, **F** The BC cells were treated as described in (**A**); caspase-3 activity was analyzed using an EnzChek Caspase-3 assay kit (*N* = 6). **G**, **H** Cell apoptosis analyses were performed using flow cytometry with FITC-conjugated Annexin V and PI staining (*N* = 4). All data are expressed as means ± SDs in triplicate samples. *P < 0.05, **P < 0.01, ***P < 0.001, and ****P < 0.0001 relative to the control group; ^#^*P* < 0.05, ^##^*P* < 0.01, ^###^*P* < 0.001, and ^####^*P* < 0.0001 relative to the cisplatin group.

Cisplatin, a first-line chemotherapeutic drug, activates the protective function of autophagy, which in turn seriously reduces the effectiveness of chemotherapy in BC cells [8]. To overcome this issue, we identified the potential of hsa-miR-30a in blocking cisplatin-related autophagy induction. First, cisplatin-induced LC3-II was suppressed by hsa-miR-30a-3p (Fig. 7C, D). Combinative treatment using cisplatin and hsa-miR-30a-3p enhanced cisplatin-induced apoptotic cell death, evident in cell morphology under a bright field microscope (Fig. 7A, B) and the downregulation of cell viability and survival (Figure S3A–D) as well as the upregulation of caspase-3 and PARP cleavage (Fig. 7C, D). Moreover, hsa-miR-30a-3p advanced cisplatin-induced caspase-3 activity and cell apoptosis (Fig. 7E–H). In conclusion, hsa-miR-30a-3p diminishes cisplatin-related protective autophagy and thus enhances the chemosensitivity of BC cells to cisplatin.

### Administration of cisplatin and hsa-miR-30a-3p considerably suppresses tumor growth

**Fig. 8:**
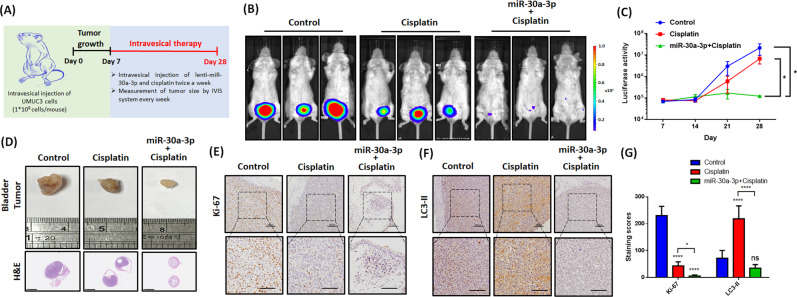
Administration of cisplatin and hsa-miR-30a-3p significantly reduce tumor growth. **A** Flowchart showing an in vivo tumor-xenograft mouse model. Luciferase-positive BC UMUC3 cells (1 × 10^6^ cells) were injected into the bladders of SCID mice. After 1 week, the mice intravesically received twice-weekly lenti-miR-30a-3p plasmid and cisplatin (2.5 mg/kg) for 4 weeks. **B**, **C** Bioluminescent images using IVIS spectrum were performed weekly, and the luminescent intensity of photons emitted from each tumor in the images was quantified (*N* = 3). **D** Tumor images representing excised tumors from each group and histologic sections of tumor were stained with H&E. **E**–**G** IHC staining exhibited Ki-67 and LC3-II expression in murine tumor tissues (*N* = 3). All data are expressed as means ± SDs in triplicate samples. *P < 0.05 relative to the control group.

Next, we addressed whether hsa-miR-30a-3p can enhance the therapeutic efficacy of cisplatin in the tumor-xenograft mouse model. SCID mice intravesically bearing a BC cell-xenografted tumor were pretreated with lenti-miR-30a-3p followed by cisplatin conduction (2.5 mg/kg) twice a week (Fig. 8A). Treatments with cisplatin alone slightly reduced tumor growth, but the combination of cisplatin with lenti-miR-30a-3p suppressed tumor growth significantly (Fig. 8B–D). IHC staining analysis revealed that after combinatory treatment with cisplatin and lenti-miR-30a-3p for 28 days, the expression of proliferation marker Ki-67 decreased in the xenograft tumor tissues (Fig. 8E, G). Furthermore, LC3-II expression induction by cisplatin was suppressed after cotreatment with lenti-miR-30a-3p (Fig. 8F, G). Together, these findings support the therapeutic potential of hsa-miR-30a-3p in enhancing the clinical benefits of cisplatin through the reduction of protective autophagy in BC.

## Discussion

Several studies have demonstrated the crucial role of autophagy in cancer, suggesting its potential in cancer treatment [28, 29]. In addition, cisplatin-treated BC cells exhibit increased autophagy activity that impedes apoptotic cell death and eventually reduces chemosensitivity [8]. The current in vitro and in vivo results demonstrate that hsa-miR-30a-3p inhibited protective autophagy through ATG5, ATG12, and Beclin 1 suppression and resulted in greater sensitivity to cisplatin, leading to improved clinical outcomes.

A comprehensive treatment approach involving surgery combined with chemoradiotherapy, radiotherapy, or immunotherapy is a first-line therapeutic option for patients with BC [30]. Recent studies have indicated that chemotherapy and radiotherapy contribute to protective autophagy in BC [8, 31]. Hwang et al. reported that cisplatin-induced autophagy is mediated by Beclin 1 in BC cells. Combinative treatment using cisplatin and autophagy inhibitors (bafilomycin A1 and chloroquine) potentially overcomes cisplatin resistance related to autophagy induction [8]. Wang et al. found that chloroquine weakened the repair of radiation-induced DNA damage and enhanced radiation-mediated cell apoptosis through caspase-3 activation. Furthermore, subcutaneous xenograft tumors demonstrated that the combination of radiation and chloroquine could impede tumorigenesis in vivo [31]. These results suggested that combining autophagy inhibitors with chemotherapy or radiotherapy is a novel and promising strategy for curing BC. In the present study, we investigated the prime mechanism by which miR-30a-3p affects BC through the inhibition of autophagy. Treatment of BC cells with the miR-30a-3p mimic significantly suppressed ATG5/12 and Beclin 1. Moreover, we found that combining the treatment of miR-30a-3p and cisplatin reduced LC3-II levels compared with cisplatin treatment alone, confirming that miR-30a-3p inhibited cisplatin-related autophagy induction. Moreover, combined treatment increased cell apoptosis in vitro and reduced tumor growth in vivo. Thus, through autophagy inhibition, miR-30a-3p is a potentially promising chemotherapeutic sensitizer in the treatment of BC by using cisplatin. In addition to cisplatin, chemotherapeutic agents include adriamycin, mitomycin C, and methotrexate. Whether miR-30a-3p has a similar antiautophagy function to those of these agents in BC requires further investigation.

Accumulating evidence exists on autophagy regulation by miRNAs through their effects on various autophagy regulatory proteins that function at the autophagy induction, vesicle nucleation, vesicle elongation, retrieval, and fusion stages [32–34]. For instance, miR-7 inhibits autophagy through the upregulation of LKB1/AMPK/mTOR signaling and the direct targeting of the stages of autophagy induction and vesicle elongation to reduce the supply of intracellular glucose to glycolysis metabolism in pancreatic cancer. Furthermore, miR-7 impedes the proliferation of pancreatic cancer cells in vitro and lung metastasis in vivo [34]. In the present study, miR-30a-3p directly targeted Beclin 1 and ATG5/12, which separately participate in the regulation of vesicle nucleation and elongation, leading to increased chemosensitivity and impaired tumor progression. Notably, miR-30b and miR-30a have a similar function in inhibiting Beclin 1 expression in vascular smooth muscle cells [35]. However, unlike miR-30a, the levels of miR-30b expression do not differ between normal and BC tissue according to the analysis of the TCGA-BC dataset, indicating that miR-30b does not serve as a specific autophagy-related miRNA in human BC. The miR-34a was found to block pathologic autophagy [36] and counteract cancer progression in a phase I clinical trial (NCT01829971) in several solid and hematological malignancies [37, 38]. The antitumor therapeutic effects of autophagy-related miRNAs must be explored in multiple clinical trials to strengthen the preclinical evidence base.

New strategies for managing MIBC are urgently required because MIBC has a high risk of recurrence and poor survival [39]. MMP2 and MMP9, which degrade type IV collagen and gelatin substrates, are highly associated with tumor dissemination, angiogenesis, and invasiveness in various solid tumor types [40, 41]. Sanaa Eissa et al. [42] found that the levels of MMP2 and MMP9 in urine samples of patients with BC increased by 4.38- and 7.13-fold in the malignant and benign groups, respectively. Our findings further demonstrate that high MMP2 expression in patients with BC was correlated with poor OS. MMP2 and MMP9 were highly expressed in the MIBC tissue compared with carcinoma in situ tissue, suggesting that MMP2 and MMP9 are diagnostic markers for detecting MIBC. Inhibiting MMP2 and MMP9 expression through miR-30a-3p reduces cell migration and invasion as well as muscle invasion in vivo. Interestingly, miR-30a-3p does not suppress MMP2 and MMP9 expression by directly binding to their 3’UTR region. The previous studies found that miRNAs might negatively regulate MMP2 and MMP9 expression by targeting upstream mediators such as Sine oculis homeobox homolog 1 [43] and AU-rich element/poly(U)-binding/degradation factor 1 [44]. These aspects deserve further investigation in BC.

**Fig. 9:**
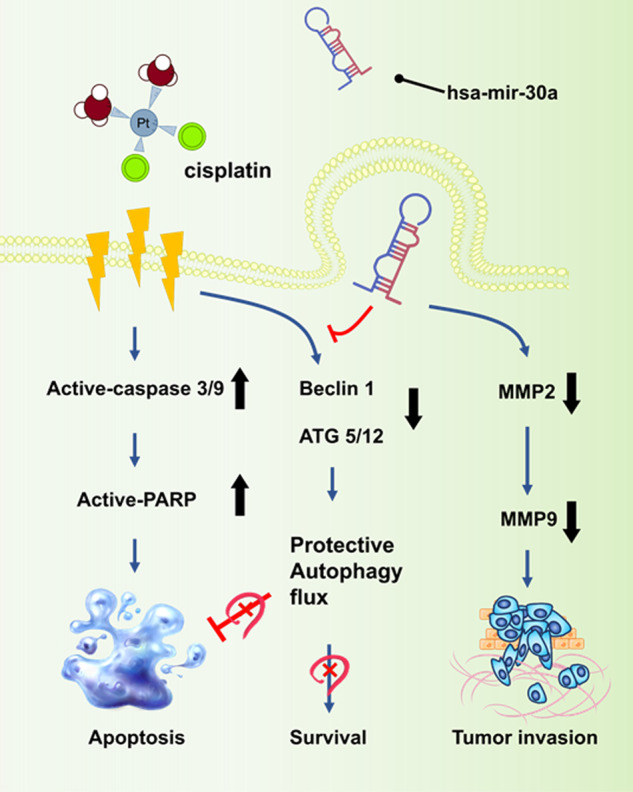
Proposed model of the clinical benefits of hsa-miR-30a-3p in BC treatment. Cisplatin is a first-line chemotherapeutic drug in BC and has antitumor effects in promoting cell apoptosis; however, its function of triggering protective autophagy is concerning in BC treatment [8]. In combination with miR-30a-3p treatment, the reduction in MMP2 and MMP9 leads to the suppression of cell migration and invasion abilities in vitro and muscle invasion in vivo. Furthermore, miR-30a-3p directly suppresses ATG5/12 and Beclin 1 expression. Cotreatment with lenti-miR-30a-3p can circumvent the induction of cisplatin-mediated protective autophagy, thus enhancing the chemosensitivity of BC to cisplatin. In sum, miR-30a-3p may serve as a potential tool of miRNA-based cancer therapy in BC treatment.

In conclusion, our study results elucidate that miR-30a-3p intervention affects BC in two manners: [1] by directly targeting ATG5, ATG12, and Beclin 1 to impair cisplatin-induced protective autophagy and leading to slow tumor growth and [2] by reducing the expression of MMP2 and MMP9 in tumors, thus inhibiting cell invasiveness in vitro and muscle invasion in vivo (Fig. 9). These findings indicate that miR-30a-3p is a tool for miRNA-based cancer therapy in patients with BC.

## Supplementary information

Supplementary Figures

Reproducibility checklist

Original Data File

## Data Availability

The data that support the findings of this study are available from the corresponding author upon reasonable request.
