# Correction: Hsa-miR-30a-3p overcomes the acquired protective autophagy of bladder cancer in chemotherapy and suppresses tumor growth and muscle invasion

**DOI:** 10.1038/s41419-022-04889-4

**Published:** 2022-05-31

**Authors:** Thomas I-Sheng Hwang, Po-Chun Chen, Te-Fu Tsai, Ji-Fan Lin, Kuang-Yu Chou, Chao-Yen Ho, Hung-En Chen, An-Chen Chang

**Affiliations:** 1 Division of Urology, Department of Surgery, Shin Kong Wu Ho-Su Memorial Hospital, Taipei, Taiwan; 2 Division of Urology, School of Medicine, Fu-Jen Catholic University, New Taipei, Taiwan; 3 Department of Urology, Taipei Medical University, Taipei, Taiwan; 4 Department of Life Science, National Taiwan Normal University, Taipei, Taiwan; 5 Translational Medicine Center, Shin Kong Wu Ho-Su Memorial Hospital, Taipei, Taiwan; 6 Department of Medical Research, China Medical University Hospital, China Medical University, Taichung, Taiwan; 7 Institute of Traditional Medicine, School of Medicine, National Yang Ming Chiao Tung University, Taipei, Taiwan

**Keywords:** Bladder cancer, Macroautophagy

Correction to: *Cell Death & Disease* 10.1038/s41419-022-04791-z, published online 21 April 2022

The original version of this article unfortunately contained an error in an author affiliation. An-Chen Chang’s correct affiliation should be Translational Medicine Center, Shin Kong Wu Ho-Su Memorial Hospital, Taipei, Taiwan. We apologize for this error. The original article has been corrected.

