# Supplementary material for: Hsa-miR-30a-3p overcomes the acquired protective autophagy of bladder cancer in chemotherapy and suppresses tumor growth and muscle invasion
Source: Cell Death Dis. 2022 Apr 21;13(4):390. doi: 10.1038/s41419-022-04791-z (PMC9023440; doi:10.1038/s41419-022-04791-z)
Supplement: Supplementary file 1 — Supplementary Figures [file 41419_2022_4791_MOESM1_ESM.docx]

**Supplementary data**

**Figure S1**


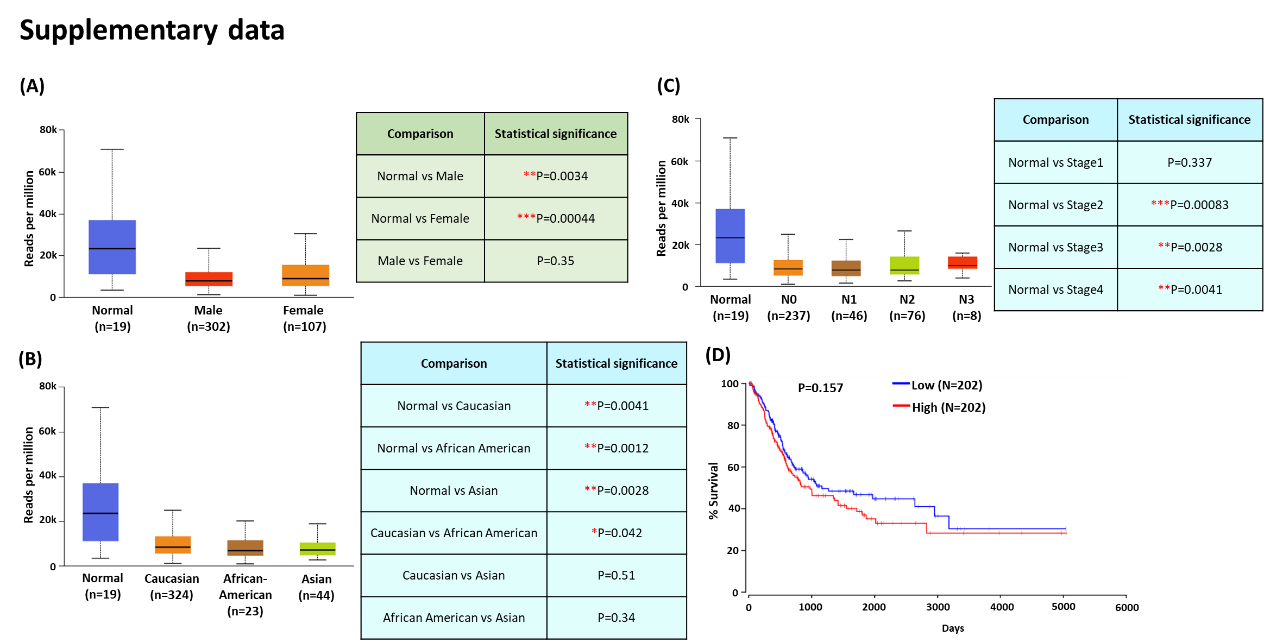


**Figure S1. UALCAN and OncoLnc analysis of BLCA samples based on the TCGA-BC dataset.** (A, B) Expression of hsa-mir-30a for different gender, and races status of BLCA samples. (C) Expression of has-miR-30a in BC tissue based on nodal metastasis status. (D) OS rates in patients with BC with high and low hsa-miR-30a expression levels were analyzed using the OncoLnc web server. All data are expressed as the mean ± the SD of triplicate samples. *P < 0.05, **P < 0.01, ***P < 0.001 compared with the normal group.

**Figure S2**


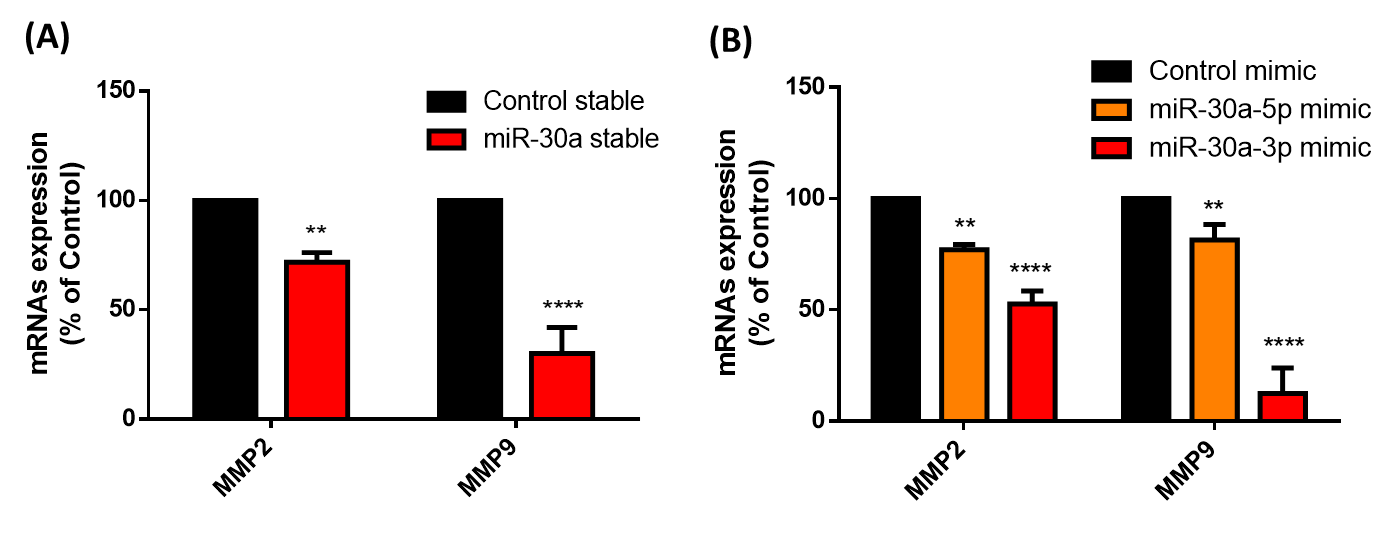


**Figure S2. Hsa-miR-30a inhibits MMP2 and MMP9 mRNA expression.** (A) The levels of MMP2 and MMP9 mRNA expression in stable BC cells were evaluated using a qRT-PCR assay. (B) Transfection of BC cells with the control mimic (25 nM), miR-30a-5p mimic (25 nM), or miR-30a-3p mimic (25 nM) for 24 h; the MMP2 and MMP9 mRNA expression levels were analyzed through qRT-PCR assay. All data are expressed as the mean ± the SD of triplicate samples. *P < 0.05, **P < 0.01, ***P < 0.001, ****P < 0.0001 compared with the normal group.

**Figure S3**


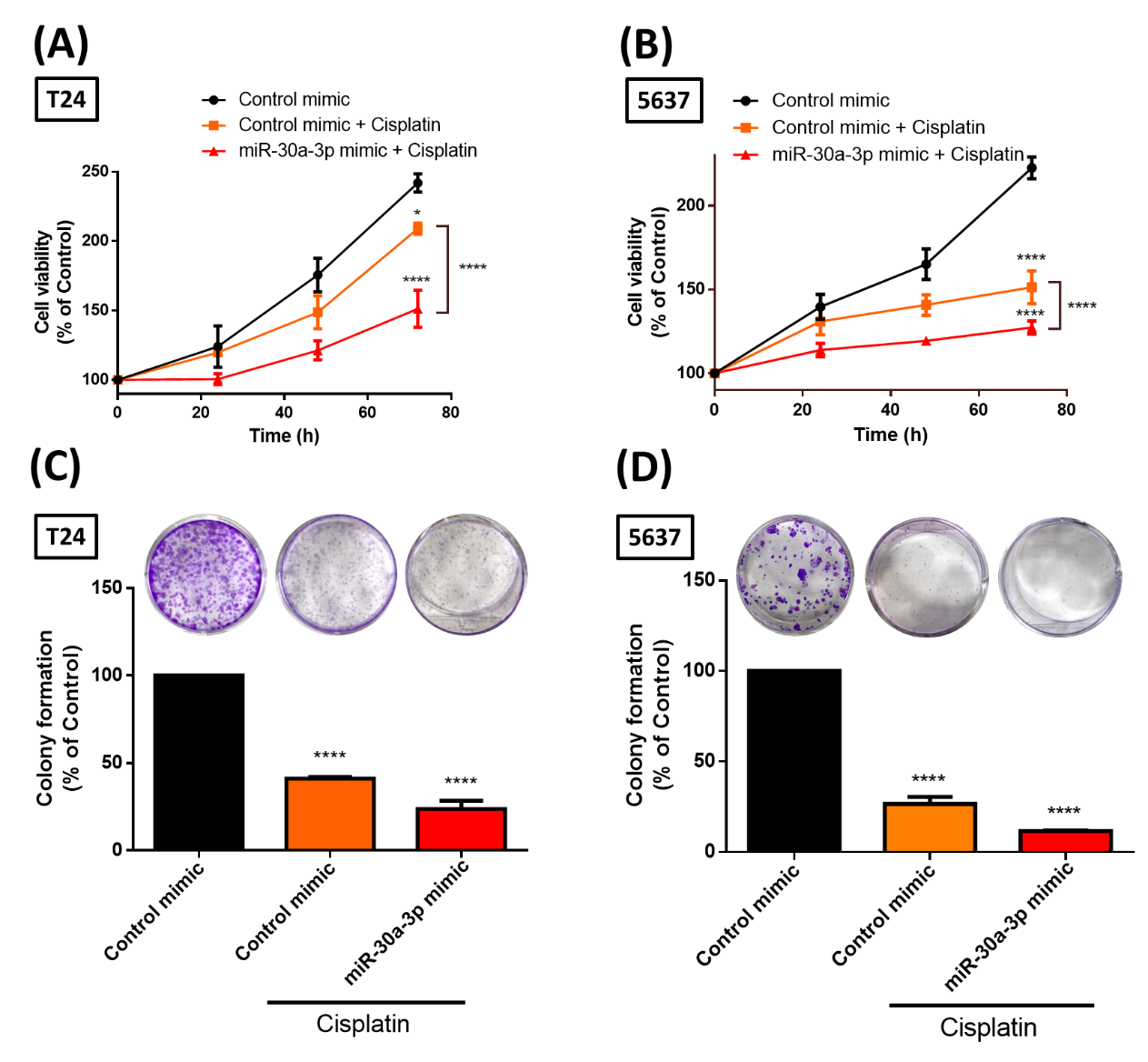


**Figure S3.** **Combined treatment with miR-30a-3p and cisplatin enhances the chemosensitivity in BC cells.**

(A, B) Transfection of BC cells with the control mimic, miR-30a-5p mimic, or miR-30a-3p mimic followed by with or without cisplatin (2 μM) incubation for 24 h. After the treatment, resazurin-based cell viability was measured. (C, D) Cell survival was detected by clonogenic survival assay. All data are expressed as the mean ± the SD of triplicate samples. *P < 0.05, **P < 0.01, ***P < 0.001, ****P < 0.0001 compared with the normal group.
