# Supplementary figures and images for: Hsa-miR-30a-3p overcomes the acquired protective autophagy of bladder cancer in chemotherapy and suppresses tumor growth and muscle invasion
Source: Cell Death Dis. 2022 Apr 21;13(4):390. doi: 10.1038/s41419-022-04791-z (PMC9023440; doi:10.1038/s41419-022-04791-z)

**Figure 2**

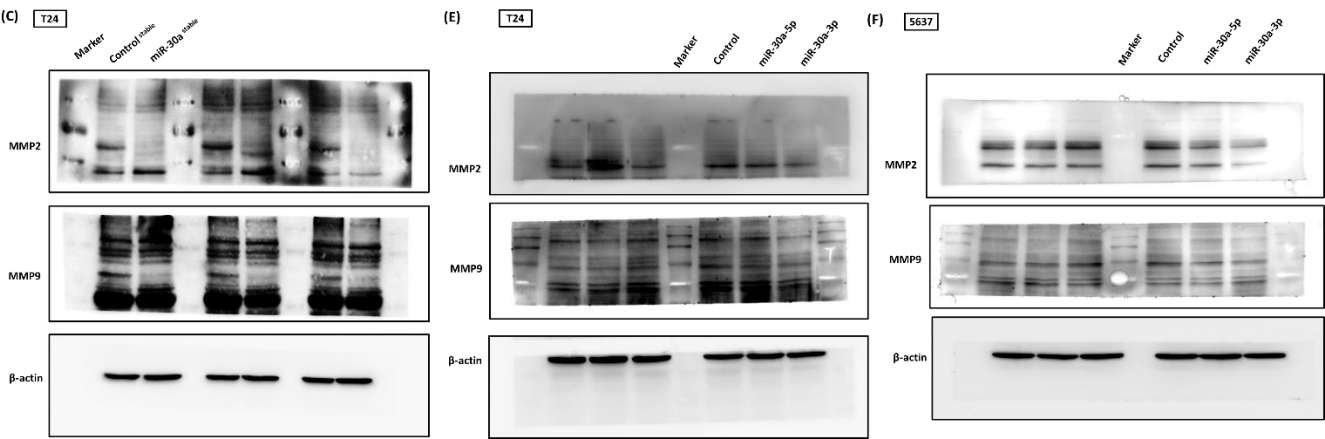

**Figure 4**

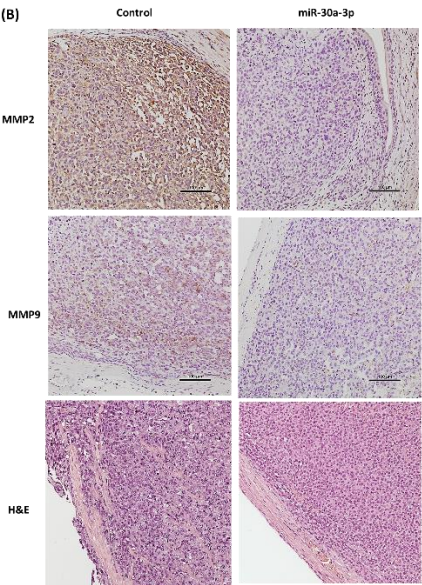

**Figure 5**

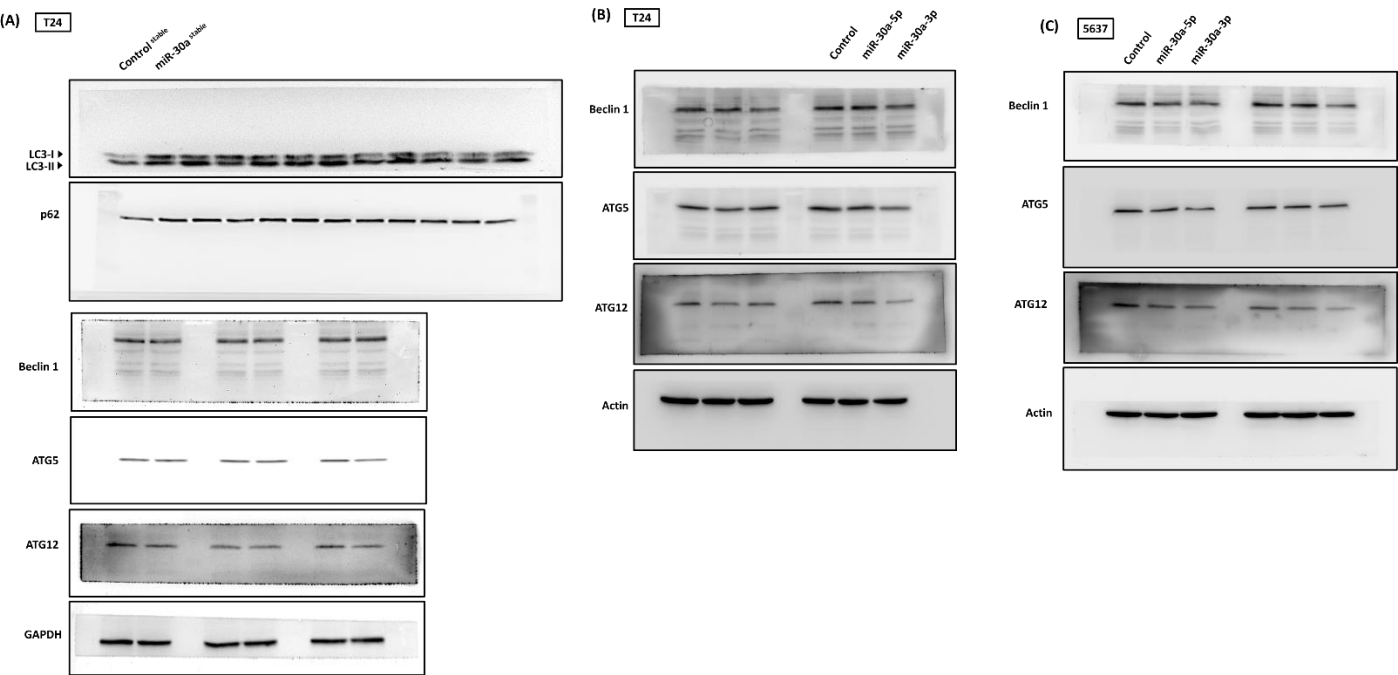

Figure 5

(G)

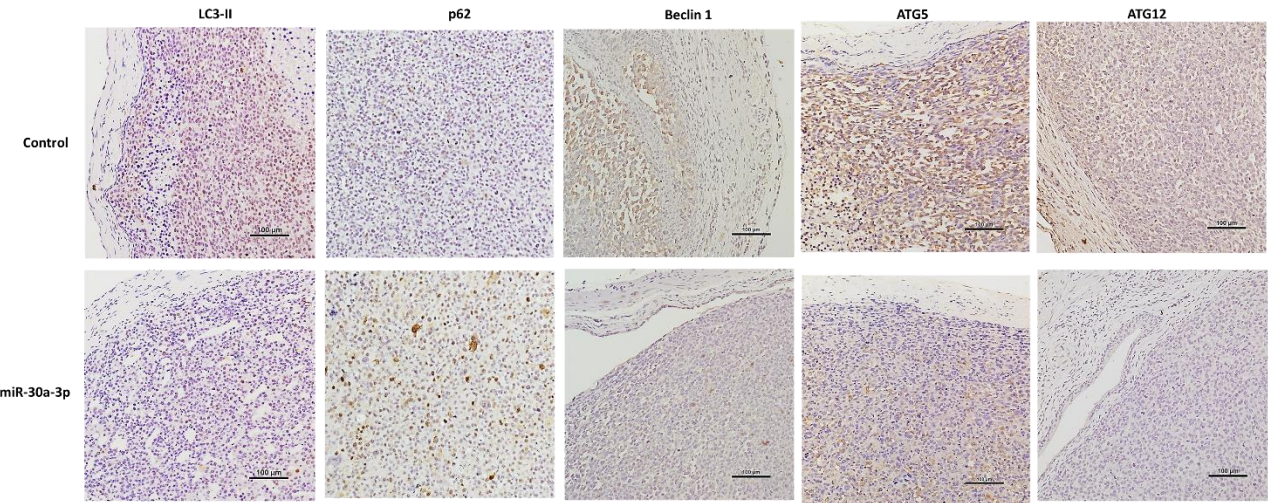

Figure 6

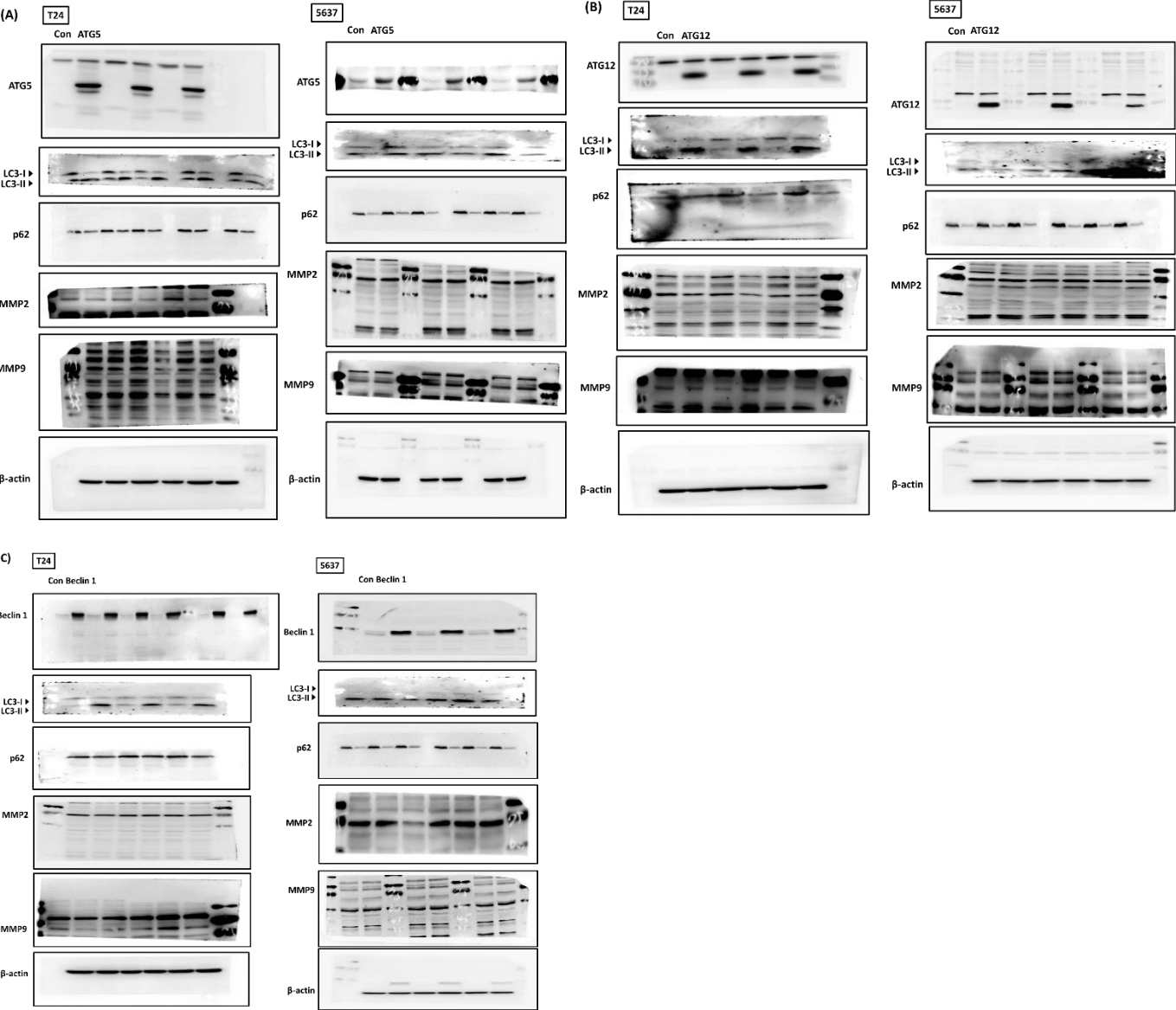

Figure 7

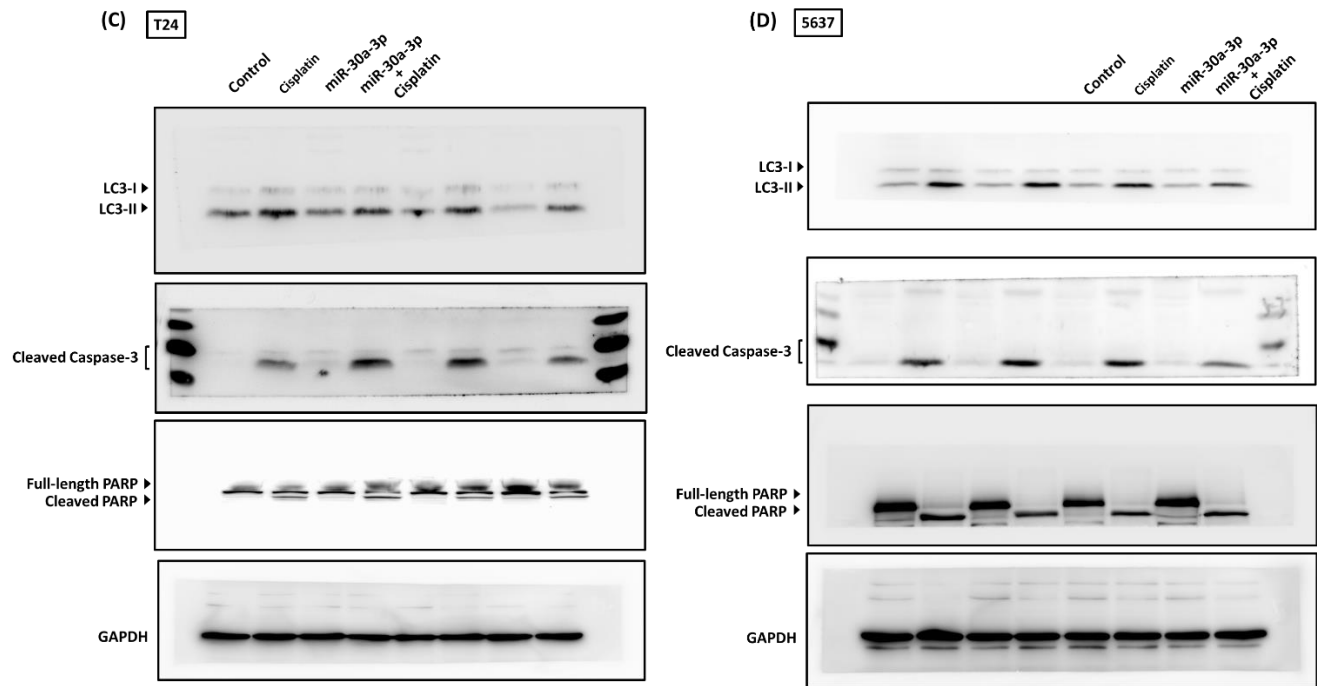

Figure 8

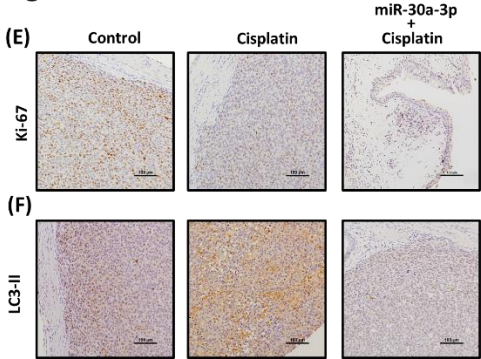

Supplement: Supplementary file 3 — Original Data File [file 41419_2022_4791_MOESM3_ESM.pdf]
